# Melatonin Regulates Chloroplast Protein Quality Control via a Mitogen-Activated Protein Kinase Signaling Pathway

**DOI:** 10.3390/antiox10040511

**Published:** 2021-03-25

**Authors:** Hyoung Yool Lee, Kyoungwhan Back

**Affiliations:** Department of Biotechnology, College of Agriculture and Life Sciences, Chonnam National University, Gwangju 61186, Korea; xanthine@naver.com

**Keywords:** melatonin, starch synthesis, MAPK kinase pathway, Clp protease, ROS defense

## Abstract

Serotonin *N*-acetyltransferase 1 (SNAT1), the penultimate enzyme for melatonin biosynthesis has shown *N*-acetyltransferase activity toward multiple substrates, including histones, serotonin, and plastid proteins. Under two different light conditions such as 50 or 100 μmol m^−2^ s^−1^, a *SNAT1*-knockout (*snat1*) mutant of *Arabidopsis thaliana* ecotype Columbia (Col-0) exhibited small size phenotypes relative over wild-type (WT) Arabidopsis Col-0. Of note, the small phenotype is stronger when growing at the 50 μmol m^−2^ s^−1^, exhibiting a dwarfism phenotype and delayed flowering. The *snat1* Arabidopsis Col-0 accumulated less starch than the WT Col-0. Moreover, *snat1* exhibited lower Lhcb1, Lhcb4, and RBCL protein levels, compared with the WT Col-0, but no changes in the corresponding transcripts, suggesting the involvement of melatonin in chloroplast protein quality control (CPQC). Accordingly, caseinolytic protease (Clp) and chloroplast heat shock proteins (CpHSPs), two key proteins involved in CPQC, as well as ROS defense were suppressed in *snat1*. In contrast, exogenous melatonin treatment induced expression of *Clp*, *CpHSP*, *APX1*, and *GST*, but not other growth-related genes such as *DWF4*, *KS*, and *IAA1*. Finally, the induction of *ClpR1*, *APX1*, and *GST1* in response to melatonin was inhibited in the mitogen-activated protein kinase (MAPK) knockdown Arabidopsis (*mpk3/6*), suggesting that melatonin-mediated CPQC was mediated, in part, by the MAPK signaling cascade. These results suggest that melatonin is involved in CPQC, which plays a pivotal role in starch synthesis in plants.

## 1. Introduction

Serotonin *N*-acetyltransferase (SNAT1) protein was first identified in Arabidopsis that interacts with the nuclear shuttle protein (NSP) of geminivirus [1]. SNAT1 belongs to the GCN5-like N-acetyltransferase (GNAT) family and is responsible for acetylation of H2A and H3 histones and the geminivirus coat protein, but not NSP [1]. Thus, SNAT1 was first called by the name of nuclear shuttle interacting (NSI) protein and reported to be involved in viral infection and pathogenicity by regulating nuclear export of the viral genome via interactions with NSP [2]. In contrast to the initial nuclear protein acetylation activity of SNAT1, the cDNA expression analyses of 31 rice *GNAT* genes in *Escherichia coli* revealed that SNAT1 also possesses serotonin *N*-acetyltransferase (SNAT) activity, catalyzing serotonin into *N*-acetylserotonin the penultimate substrate for melatonin biosynthesis [3,4]. SNAT1 exhibited *K*_m_ and *V*_max_ values for serotonin of 309 μM and 1.4 nmol/min/mg protein, respectively, and was localized to chloroplasts but in a different manner to previous nuclear localization results for NSI [1,4].

Recently, it was reported that SNAT1 was also discovered to catalyze the *N*-acetylation of chloroplast proteins, such as Lhcb1.4, which resulted in an impaired state transition in a *SNAT1*-knockout Arabidopsis (*snat1*) [5]. In addition to the state transition changes, many other biochemical phenotypes have been observed in the *snat1* Arabidopsis, including enhanced pathogen susceptibility [6], reduced high-light stress tolerance [7], salinity hypersensitivity [8], and increased endoplasmic reticulum stress susceptibility [9]. These many defects in *snat1* Arabidopsis have been complemented by exogenous supplementation of melatonin, suggesting that melatonin is predominantly responsible for the many phenotypes induced by *SNAT1* knockout rather than the acetylation of chloroplast proteins. Correspondingly, the *snat1* decreased melatonin by 25% compared to the wild type (5), and its overexpression line (OE) increased melatonin two-fold relative to the wild type (7).

The major genes responsible for melatonin biosynthesis are tryptophan decarboxylase, which catalyzes tryptophan into tryptamine, and tryptamine 5-hydroxylase, which converts tryptamine into serotonin. SNAT plays a pivotal role in synthesizing *N*-acetylserotonin, the key substrate for melatonin biosynthesis, and *N*-acetylserotonin is then converted into melatonin by *N*-acetylserotonin O-methyltransferase [10]. Although *SNAT* exists as multiple isogenes in plants and belongs to the GNAT family, these isogenes share a very low amino acid identity, except for within the acetyltransferase domain. *SNAT1* is derived from cyanobacteria, since cyanobacteria also harbor a *SNAT1* orthologous gene, and it shares a 58% amino acid identity with that of rice *SNAT1* [11]. Furthermore, the *SNAT1* ortholog of red algae resides in the chloroplast genome, whereas those of plants and green algae reside in the nuclear genome [12]. This suggests that *SNAT1* was transferred from the chloroplast to the nuclear genome during plant evolution, and it may have specific roles in chloroplast functionality, such as the state transition in photosynthesis [5]. Overall, *SNAT1* plays a pivotal role in melatonin biosynthesis in many plant species, including rice. Its overexpression (OE) leads to increased melatonin production, whereas its suppression results in decreased melatonin production and the corresponding physiological and biochemical phenotypes described above [13,14,15,16].

Melatonin had long been recognized as an animal pineal hormone that regulates many physiological activities such as sleep, the circadian rhythm, innate immunity, and cellular oxidative status [17]. However, melatonin has also been identified in various plants [18,19], where it has pleiotropic biological roles in plant growth and development, and in plant defense systems against biotic and abiotic stresses [20]. The representative roles of melatonin in plant growth and development include promoting seedling growth [13], early flowering [21,22], enhanced seed germination and viability [23,24], delayed senescence [25], diurnal stomatal closure [26], and increased secondary metabolites [27], etc. [20,28,29]. Moreover, melatonin has a profound effect on plant defenses against a vast array of adverse environmental stresses, helping plants to survive and thrive [30]. For example, melatonin confers tolerance in response to virus and pathogen attacks [31,32], and to many abiotic stresses, including cold, heat, salinity, drought, heavy metals, herbicides, and tunicamycin [9,20,33], via either its potent antioxidant role or melatonin signaling cascade through its receptor [34,35].

Although the multiple roles of melatonin against various stresses have been investigated extensively in plants, its role in chloroplast function during normal plant growth and development has not been examined in detail. In this report, we used a *snat1* Arabidopsis to show that changes in melatonin biosynthesis result in the *snat1* phenotype, i.e., dwarfism, reduced starch synthesis, and delayed flowering in conjunction with an impaired chloroplast quality control by way of MAPK cascade.

## 2. Materials and Methods

### 2.1. Plant Material and Growth Conditions

All *Arabidopsis thaliana* lines used in this study were in the ecotype Columbia (Col-0) background. *SNAT1* (At1g32070) knockout Arabidopsis line containing T-DNA insert in SALK_020577 (*snat1*) was obtained from the Arabidopsis Biological Resource Center (Ohio State University, Columbus, OH, USA), as described previously [7]. Transgenic lines overexpressing *SNAT1* (OE) and double suppression lines of *MPK*3 and *MPK6* (*mpk3/6*) have been previously described [7,36]. Plants were grown in plastic pots containing a commercial horticultural substrate (coco peat (47%): peat moss (35): vermiculite (10): zeolite (7%) (Farmhannong, Seoul, Korea)): perlite (3:1) (SJ Company, Ulsan, Korea). The soil mixture was washed out by two-times irrigation with tap water to exclude possible interference of artificial nutrition. Controlled environmental conditions were provided in a growth room at 23 °C and relative humidity of 50% under 12-h light/12-h dark photoperiod with white light illumination (50 μmol m^−2^ s^−1^). Fluorescent light from OSRAM (Seoul, Korea) with 50:50 of 6500K (865 FPL36EX-D) and 4000K (840 FPL36EX-W) was used for the light source.

### 2.2. Flowering Time, Leaf Area, and Weight Measurements

Flowering times were determined by counting the number of rosette or cauline leaves on the main shoot when the plants had the first flower at about seven weeks after seeding. At the same time, rosette leaf area was measured using Fiji ImageJ, as described previously [37]. Photographs of rosette leaves were taken next to a ruler that was used as a reference to convert pixels to the corresponding metric unit. Data were processed using Microsoft Excel 2010. The fresh weight of aerial parts was measured at indicated growth stages. At least five plants per genotype were used for each measurement, and average and standard deviation were calculated accordingly.

### 2.3. Starch Staining

Iodine-stained visualization of starch was conducted as described [38]. Briefly, whole Arabidopsis rosettes of four- or six-week-old plants were collected and decolorized using 90% (*v/v*) of hot ethanol. Ethanol was removed by rinsing in water before staining Lugol’s iodine Reagent (Sigma-Aldrich, Saint-Louis, MO, USA). Over 6-h iodine staining, plants were destained in water until optimal visualization of the amylose–iodine complex in starch was achieved.

### 2.4. Melatonin Treatment

All transcripts of target genes studied here have been positively regulated during daylight. To abstain from the daylight induction of these genes, four-week-old Arabidopsis plants grown under the standard condition (50 μmol m^−2^ s^−1^) were infiltrated with 1 μM melatonin (in 2 mM MgCl_2_) by needless 1 mL syringe at ZT0 and transferred to the dim light condition (7 μmol m^−2^ s^−1^), followed by sample harvest at various time intervals. Melatonin was infiltrated into the abaxial sides of four-week-old rosette leaves of Arabidopsis.

### 2.5. Protein Extraction and Protein Gel Blotting Analysis

Protein extracts were prepared with 40 mM HEPES, pH7.5, 100 mM NaCl, 1 mM EDTA, 10% glycerol, 0.2% Triton X-100, and 1x Roche Protease Inhibitor Cocktail (Roche Applied Science, Indianapolis, IN, USA) and then centrifuged at 10,000× *g* for 10 min at 4 ℃. Aliquots of the supernatant were mixed with sample buffer (Tris-HCL, pH 6.8, 10% SDS, 10 mM DTT, 20% glycerol, and 0.05% bromophenol blue). Then, samples were boiled and loaded onto SDS–PAGE gels. After electroblotting on a nitrocellulose membrane, protein gel blot analysis was performed using antibodies against Lhcb1, Lhcb4, RBCL, RBCS and ClpR1 (Agrisera AB, Vannas, Sweden). Secondary rabbit antibody conjugated with Horseradish Peroxidase (Roche) was incubated with the membrane for at least 1 h. Proteins were detected using the ECL system (RPN2132; Amersham Biosciences, Piscataway, NJ, USA).

### 2.6. RNA Analysis

Total RNA was extracted from Arabidopsis plants using a Nucleospin RNA Plant Kit (Macherey-Nagel, Duren, Germany). Reverse transcription was performed using a Stratagene Reverse Transcription Kit (Stratagene, La Jolla, CA, USA) with 500 ng of oligo (dT)18 or random octamer primer for *RBCL* and *ClpP1* (CancerROP, Seoul, Korea). The PCR reaction was conducted as following conditions initial denaturation 95 °C (3 min), denaturation 95 °C (30 s), annealing 56 °C (30 s), and extension 72 °C (1 min) with 30 µL of master mix. qRT-PCR was performed on a Mic qPCR Cycler System (Bio Molecular Systems, Queensland, Australia) using SYBR Green RT-PCR Reagent Kit (Luna Universal qPCR Master Mix; NEB, Hitchin, UK) in accordance with the manufacturer’s protocol. The primer sequences for RNA analysis are shown in Table S1. *EF-1α* (*EF1ALPHA*; At5g60390) was used for signal normalization. The data were analyzed by analysis of variance using IBM SPSS Statistics 25 software (IBM Corp. Armonk, NY, USA). Means with different letters or asterisks indicate significantly different values at *p* < 0.05 according to a post hoc Tukey’s honestly significant difference (HSD) test. All data are presented as mean ± standard deviation.

## 3. Results

### 3.1. Phenotypic Features of the SNAT1-Knockout Arabidopsis Mutant

**Figure 1:**
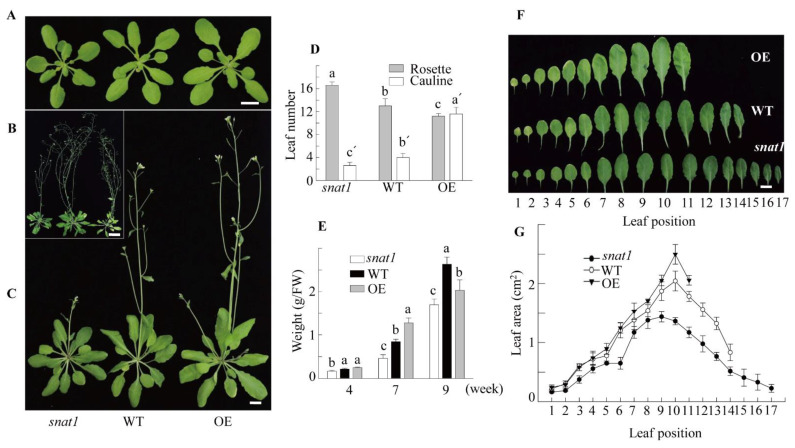
(**A**) Rosette or (**B**) flowering, (**C**) phenotype of wild type (WT) (Col-0), *snat1*, and *SNAT1* overexpression (OE) Arabidopsis lines. Plants were grown for four (**A**), nine (**B**), or seven weeks (**C**) under 50 μmol m^−2^ s^−1^ light conditions. (**D**) The flowering times of Arabidopsis were denoted as the total number of rosette and cauline leaves averaged over five independent plants. (**E**) Fresh weights of the WT, *snat1*, and OE lines 4–9 weeks after planting. (**F**) Representative rosette leaves from the WT, *snat1*, and OE lines after the OE line had started to bolt. (**G**) Rosette leaf areas of each leaf position in the WT, *snat1*, and OE lines, measured at the same time as in F. Scale bar: 1 cm (**A**,**C**,**F**) and 3 cm (**B**). Different letters indicate significant differences between groups (small letters or small letters with an apostrophe) (Tukey’s post hoc HSD test; *p* < 0.05). *SNAT1* encodes the gene with At1g32070.

The *SNAT1* knockout Arabidopsis (*snat1*) showed slight dwarfism when grown under an ambient light regime (100 μmol m^−2^ s^−1^) but exhibited a stronger dwarf phenotype under a decreased light regime (50 μmol m^−2^ s^−1^) (Figure S1). A light intensity of 50 μmol m^−2^ s^−1^ was sufficient for normal growth and development of *Arabidopsis thaliana* ecotype Col-0, as indicated below. Under a light intensity of 50 μmol m^−2^ s^−1^, WT Arabidopsis Col-0 showed healthy growth (Figure 1), whereas the *snat1* showed the dwarfism phenotype and delayed flowering (Figure 1A–C). In contrast, *SNAT1* OE resulted in faster growth and earlier flowering compared with the WT, but at nine weeks, the total biomass was lower than that of the WT (Figure 1A–C). Compared with the WT, the *snat1* had more leaves, whereas OE resulted in fewer leaves at the seven-week flowering stage (Figure 1D,F). The *snat1* had lower biomass and smaller leaf area compared with the WT (Figure 1E,G). OE resulted in higher numbers of cauline leaves compared with the WT, but the underlying mechanism for this is unclear (Figure 1D). Collectively, these growth parameters indicated that the *snat1* exhibits retarded growth and development phenotypes compared with the WT, suggesting that SNAT1 has physiological roles in growth, which were observed previously in the *SNAT1* RNAi rice seedlings [13].

### 3.2. Defective Starch Accumulation in the Snat1 Mutant

**Figure 2:**
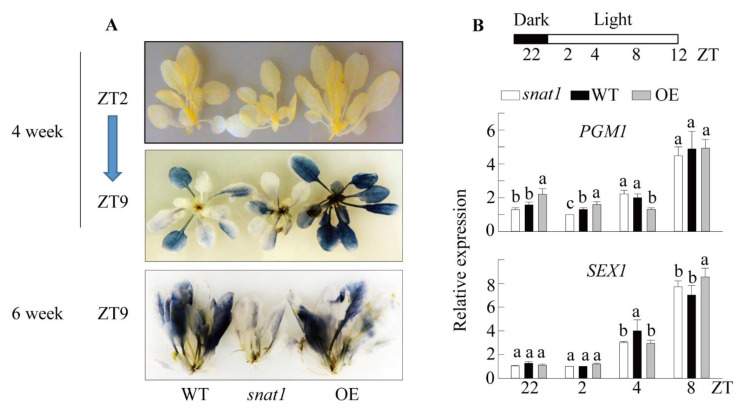
(**A**) Comparison of leaf starch contents. WT (Col-0), *snat1*, and *SNAT1* OE Arabidopsis lines grown under 50 μmol m^−2^ s^−1^ light conditions for four or six weeks were decolorized and stained with iodine solution, washed with water, and photographed. Plants were collected at Zeitgeber time (ZT)2 or ZT9. (**B**) qRT-PCR analysis showing induced expression of starch metabolism-related genes in WT (Col-0), *snat1*, and OE Arabidopsis plants. Leaf samples were collected at various ZT intervals. The target transcript levels were normalized to those of the *EF1α* endogenous control. ZT, Zeitgeber time; ZT0 represents dawn. Values are means ± standard deviation of three independent experiments. Different letters indicate significant differences (Tukey’s post hoc HSD test; *p* < 0.05).

We deduced that the growth retardation of *snat1* Arabidopsis was likely associated with the growth rate-related starch synthesis. To evaluate starch biosynthetic capacity, we measured starch levels during the day, which were increased by photosynthesis [39]. Lugol staining of plants showed undetectable starch levels at Zeitgeber time (ZT) 2 (after 2 h of light exposure) in the WT, *snat1*, and OE lines (Figure 2A). Starch accumulation was clearly evident at ZT9 in the WT but was much less evident in *snat1*. In particular, higher levels of starch accumulated in the OE than WT lines. Changes in starch accumulation during the day were equally observed in both four- and six-week-old Arabidopsis plants among WT, *snat1*, OE, providing strong evidence for the role of SNAT1 in photosynthesis. To ascertain whether the changes in starch accumulation were associated with changes in starch synthesis and degradation [40], we quantified the mRNA levels of phosphoglucomutase (*PGM1*) and phosphoglucan phosphatase (*SEX1*). As shown in Figure 2B, the diel expression patterns of these two genes were not significantly different among the WT, *snat1*, and OE plants. These data indicated that the decrease in starch synthesis in *snat1* was not due to transcript changes involved in starch synthesis and degradation.

### 3.3. Suppression of Lhcb1, Lhcb4, and RBCL Proteins in the snat1 Mutant

**Figure 3:**
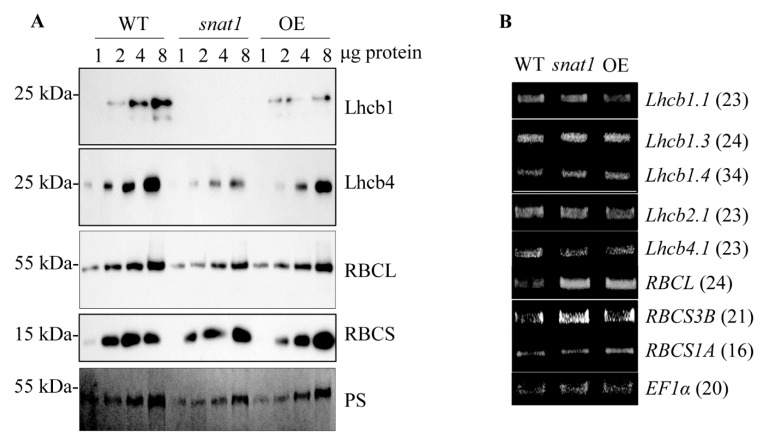
(**A**) Western blot analysis using anti-Lhcb1, -Lhcb4, -RBCS, and -RBCL antibodies in the WT, *snat1*, and OE Arabidopsis lines at ZT8. Total leaf protein extracts (1, 2, 4, and 8 µg) were subjected to 14% SDS–PAGE. The immunoblot was probed with specific antibodies, as indicated on the right. Molecular weights are shown on the left. The bottom panel shows a loading control stained with Ponceau S solution (PS). (**B**) qRT-PCR analysis of the corresponding transcripts at ZT8. *EF1α* was used as a loading control. Numbers in parentheses indicate the number of PCR cycles.

Light-harvesting antenna proteins such as Lhcb1 play important roles in photosynthetic electron transport. For example, a decrease in the Lhcb1 level reduced chlorophyll levels and state transition in Arabidopsis [41]. We performed Western blot analyses to evaluate Lhcb protein levels in *snat1*. As shown in Figure 3, the protein levels of Lhcb1 and Lhcb4 were greatly reduced in *snat*1, compared with the WT. The ribulose-1,5-bisphosphate carboxylase/oxygenase (RBC) large subunit (RBCL) was also slightly reduced in *snat1*, whereas the RBC small subunit (RBCS) was not affected, compared with the WT. The OE line of Arabidopsis also exhibited a decrease in the Lhcb1 protein level, but not to the same extent as in *snat1*. In marked contrast to the protein levels, the mRNA levels of these proteins, except for *RBCL*, were not affected, suggesting that the reduced levels of Lhcb1 and Lhcb4 in *snat1* resulted from altered protein stability in the chloroplasts rather than from altered protein synthesis in the cytoplasm.

### 3.4. Downregulation of ClpR1, a Chloroplast Molecular Chaperone, in the snat1 Mutant

**Figure 4:**
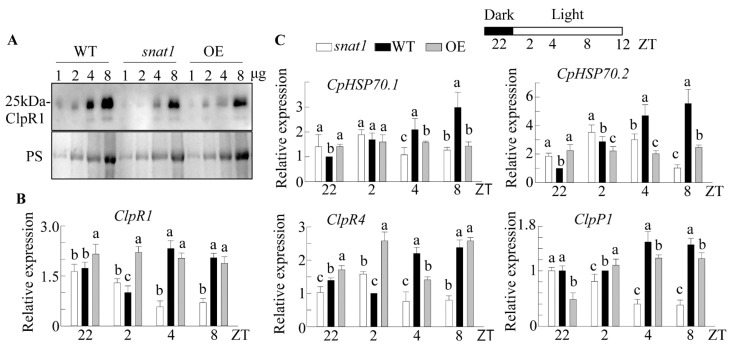
(**A**) Protein level of ClpR1 in the WT, *snat1*, and OE Arabidopsis lines at ZT8. (**B**) Diurnal expression of *ClpR1*, *ClpR4*, and *ClpP1*. (**C**) Diurnal expression of *CpHSP70.1* and *CpHSP70.2* in the WT, *snat1*, and OE lines at various ZT intervals. The relative fold expression values are normalized to *EF1α* expression. Error bars show the standard deviation of three biological replicates. The Ponceau S solution (PS) was used as a protein loading control. Different letters indicate significant differences (Tukey’s post hoc HSD test; *p* < 0.05).

For optimal functionality, the majority of chloroplast proteins are imported from the cytoplasm. In the chloroplast, these proteins undergo precise targeting and folding via the chloroplast protein quality control (CPQC) system, which is controlled by plastid chaperones and heat shock proteins (CpHSPs) [42,43]. Caseinolytic protease (Clp) is an ATP-dependent protease with multiple isoforms that functions as a molecular chaperone. The ClpR1 subunit of Clp plays an important role in chloroplast development by controlling Lhcb2 protein levels, and the *ClpR1*-knockout mutant had dwarfism and virescent phenotype [44]. Interestingly, *snat1* exhibited decreased ClpR1 protein in conjunction with decreased *ClpR1* mRNA levels, compared with the WT (Figure 4A,B). Other Clp subunits, such as *ClpR4* and *ClpP1*, were also downregulated in *snat1* compared with the WT at ZT4 and ZT8. The OE line showed a slight decrease in the ClpR1 protein level with a transit increase in the levels of *ClpR1*, *ClpR4*, and *ClpP1* mRNA at ZT2, compared with the WT. Additionally, mRNA levels of the chloroplast heat shock proteins *CpHSP70.1* and *CpHSP70.2* were significantly downregulated in *snat1*, compared with the WT (Figure 4B). The suppression of *CpHSP70.1* and *CpHSP70.2* in the OE line was unexpected, but their suppression was partly coupled with suppression of the ClpR1 protein (Figure 4A). These data suggest that suppression of the Lhcb and RBCL proteins in *snat1* is attributed to the downregulation of chloroplast chaperones such as Clp and CpHSP.

### 3.5. Downregulation of Ascorbate Peroxidase and Glutathione S-Transferase in the snat1 Mutant

**Figure 5:**
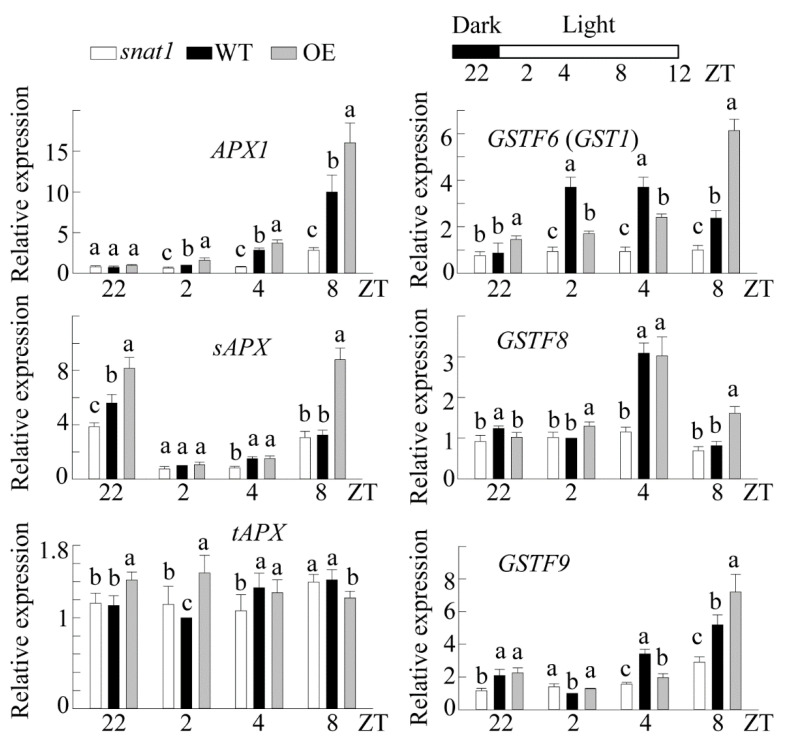
Temporal qRT-PCR analysis of various Arabidopsis genes involved in the reactive oxygen species (ROS) defense and scavenging systems at various ZT intervals. Total RNA was isolated, and transcript levels in WT, *snat1*, and OE Arabidopsis lines were measured by qRT-PCR. The relative fold expression values are normalized to *EF1α* expression. Error bars show the standard deviation of three biological replicates. Different letters indicate significant differences (Tukey’s post hoc HSD test; *p* < 0.05).

Chloroplasts are the major organelles responsible for the production of reactive oxygen species (ROS) such as O_2_^-^ and H_2_O_2_ during photosynthesis. Although ROS can cause oxidative damage, they also act as retrograde signals in chloroplasts to induce a series of nuclear-encoded genes responsible for photosynthesis-related genes such as *Lhcb* and *RBCL*, chaperone genes such as *CpHSP* and *Clp*, and antioxidant genes such as ascorbate peroxidase 1 (*APX1*) and glutathione S-transferase (*GST1*) [45]. Since melatonin is a potent antioxidant believed to be enriched in chloroplasts [46,47], it is highly likely that melatonin plays a specific role in ROS balance in plants. Thus, we investigated whether ROS-responsive genes are also modulated in *snat1* by measuring the mRNA expression levels of various APX and GST isoenzymes during the day. As shown in Figure 5, *APX1*, encoding a cytoplasmic APX with a central role in the chloroplast H_2_O_2_-scavenging system of Arabidopsis [48], was induced at ZT8 in the WT, but its induction was greatly inhibited in *snat1*. In contrast, two other *APX* isogenes—*sAPX* (stomatal and mitochondrial APX) and *tAPX* (thylakoid APX)—were not different between *snat1* and WT lines at ZT8. Interestingly, all three *GST* genes were induced during the day in the WT, but not in *snat1*. In contrast to the results for *snat1*, the OE line exhibited much higher induction of the *APX1*, *sAPX*, and all three *GST* genes compared with the WT at ZT8. This enhanced expression of *APX* and *GST* genes by OE possibly contributed to the enhanced growth and increased starch synthesis during the early growth stages (Figure 1 and Figure 2). However, a slight reduction in Lhcb1, Lhcb4, and ClpR1 protein levels in the OE line eventually led to a reduction in biomass relative to the WT at nine weeks (Figure 1). Taken together, these data suggest that SNAT1 plays a pivotal role in regulating antioxidant genes, such as *APX1*, which predominantly acts as a retrograde signal of chloroplast ROS [45]. In analogy, it was reported that singlet oxygen in chloroplasts also induced *SNAT1* and melatonin synthesis, followed by an increase in *APX1* in the *flu* knockout mutant of Arabidopsis [7].

### 3.6. Induction of Genes Responsible for the CPQC and ROS Defense Systems by Exogenous Melatonin Treatment

**Figure 6:**
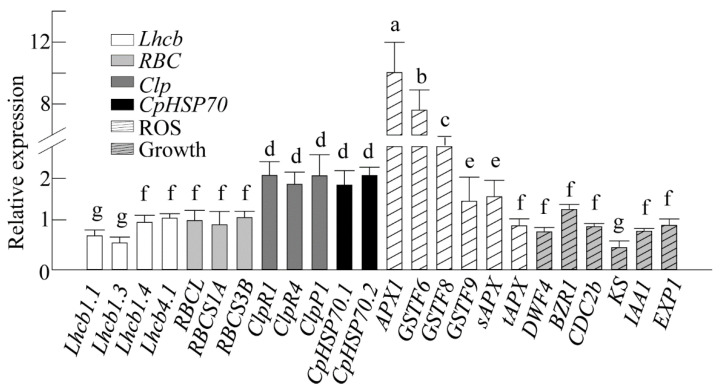
Effects of exogenous melatonin treatment on various genes related to photosynthesis, protein quality control, ROS defense, and growth. WT Arabidopsis (four weeks old) leaves were infiltrated with 1 μM melatonin at ZT0 and transferred to dim-light conditions (7 μmol m^−2^ s^−1^) to rule out potential light induction of various target genes. The samples were harvested at ZT6 for total RNA isolation. The genes evaluated were *Lhcb1.1* (At1g29910), *Lhcb1.3* (At1g29930), *Lhcb1.4* (At2g34430), *Lhcb4.1* (At5g01530), *RBCL* (AtCg00490), *RBCS1A* (At1g67090), *RBCS3B* (At5g38410), *ClpR1* (At1g49970), *ClpR4* (At4g17040), *ClpP1* (AtCg00670), *CpHSP70.1* (At4g24280), *CpHSP70.2* (At5g49910), *APX1* (At1g07890), *GSTF6* (*GST1*; At1g02930), *GSTF8* (At2g47730), *GSTF9* (At2g30860), *sAPX* (At4g08390), *tAPX* (At1g77490), *DWF4* (At3g50660), *BZR1* (At1g75080), *CDC2b* (At3g54180), *KS* (At1g79460), *IAA1* (At4g14560), and *EXP1* (At1g69530). The relative fold expression values are normalized to *EF1α* expression, and the expression level after each mock treatment (2 mM MgCl_2_) was set at a relative level of 1. Different letters indicate significant differences (Tukey’s post hoc HSD test; *p* < 0.05).

To elucidate the direct relationship between SNAT1 and melatonin in response to the CPQC and ROS defense systems, melatonin (1 μM) was infiltrated onto Arabidopsis leaves and incubated for 6 h. This was followed by qRT-PCR analysis to investigate the induction patterns of the related genes. As shown in Figure 6, there were no increases in four *Lhcb* isogenes, two *RBCS* isogenes, or *RBCL* after melatonin treatment. However, there was a two-fold increase in the expression of three *Clp* genes, such as *ClpR1*, *ClpR4*, and *ClpP1*, and two *CpHSP* genes, such as *CpHSP70.1* and *CpHSP70.2*. The highest induction was observed in *APX1* followed by *GSTF6* (*GST1*) and *GST8*, whereas *tAPX* was not induced. Other genes that affect Arabidopsis growth were also evaluated for their possible involvement in the *snat1* dwarfism phenotype. These genes were associated with brassinosteroids (*DWF4*, *BZR1*, and *CDC2b*), gibberellin biosynthesis (*KS*), and auxin responsiveness (*IAA1* and *EXP1*). These hormone-related genes were not enhanced in response to melatonin treatment. Together with the results observed in *snat1*, SNAT1-catalyzed melatonin deficiency appears responsible for the dwarfism phenotype via the regulation of genes or proteins involved in the CPQC and ROS defense systems rather than various hormonal genes related to growth.

### 3.7. Induction of ClpR1 Protein by Melatonin via the Mitogen-Activated Protein Kinase (MAPK) Signaling Pathway

**Figure 7:**
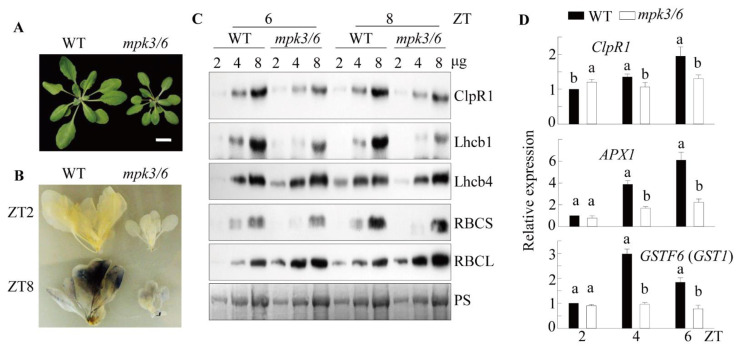
(**A**) Rosette phenotype of WT (Col-0) and *mpk3/6*-double knockdown plants. Plants were grown for four weeks under a light intensity of 50 μmol m^−2^ s^−1^. (**B**) Leaf starch contents of the WT (Col-0) and *mpk3/6* lines. Plants were collected at ZT2 or ZT8. (**C**) Western blot analysis using anti-ClpR1, -Lhcb1, -Lhcb4, -RBCS, and -RBCL antibodies, as described in Figure 3. Plants were collected at ZT6 or ZT8. (**D**) Diurnal expression of *ClpR1*, *APX1*, and *GST1* at ZT2, ZT4, and ZT6. Scale bar: 1 cm. Different letters indicate significant differences (Tukey’s post hoc HSD test; *p* < 0.05).

**Figure 8:**
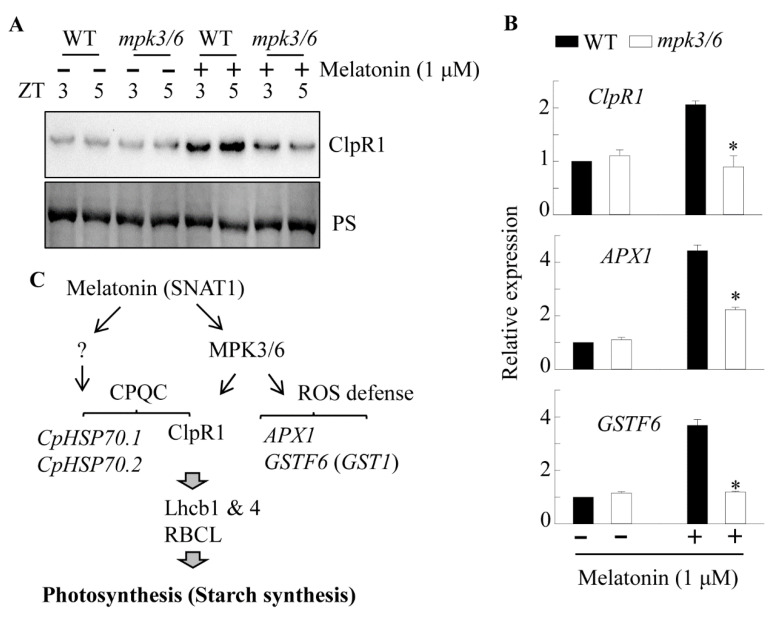
(**A**) Induction of ClpR1 in the WT and *mpk3/6*-double knockdown plants in response to melatonin. To eliminate light interference in target gene induction, Arabidopsis leaves were infiltrated with 1 μM melatonin at ZT0 and transferred to dim light (7 μmol m^−2^ s^−1^) conditions for 3 h (ZT3) and 5 h (ZT5) before sample harvest. Ponceau S stained blots were used as the loading control. (**B**) Gene expression analysis of *ClpR1, APX1*, and *GST1* after treatment with 1 μM melatonin in the WT and *mpk3/6* lines at ZT5. (**C**) Proposed model of melatonin-mediated CPQC in Arabidopsis. Asterisks denote significant differences as determined by post hoc Tukey’s HSD test at *p* < 0.05.

Melatonin-mediated defense responses against pathogens and endoplasmic reticulum (ER) stress require MAPK signaling [9,36]. ROS-mediated retrograde signaling also depends on MAPK signaling [45,49]. We used an *MPK3/MPK6*-double knockdown Arabidopsis line (*mpk3/6*) to confirm whether the MAPK pathway is involved in the CPQC and ROS defense systems [36]. The *mpk3/6* exhibited a dwarfism phenotype and a defect in starch accumulation at ZT8, comparable with that in *snat1* (Figure 7A,B). Both the ClpR1 and Lhcb1 protein levels were significantly downregulated in *mpk3/6*, whereas the Lhcb4 and RBCS protein levels were similar to those in the WT. In contrast, the RBCL level was higher in *mpk3/6* than in the WT. The different protein expression patterns in *snat1* and *mpk3/6* indicate that MPK3/6 does not function exclusively in melatonin signaling. Meanwhile, the expression of *ClpR1*, *APX1*, and *GSTF6* (*GST1*) transcripts was lower in *mpk3/6* than in the WT, suggesting defects in chloroplast chaperone activity and the ROS defense system in *mpk3/*6 (Figure 7D). Finally, to ascertain whether melatonin-induced ClpR1 was dependent on the MAPK signaling pathway, the leaves of WT and *mpk3/6* were challenged with melatonin, and the levels of ClpR1 protein and related transcripts were measured. As shown in Figure 8, ClpR1 was induced in response to 1 μM melatonin treatment in the WT but was significantly inhibited in the *mpk3/6* mutant. Moreover, induction of the *ClpR*1 transcript together with *APX1* and *GST1* was suppressed in *mpk3/6*, compared with the WT. These data suggest that melatonin is involved in the induction of ClpR1 via the MAPK signaling cascade, followed by enhanced and stable expression of Lhcb1 protein and related transcripts such as *APX1*, and *GSTF6*, which are important regulators of growth and photosynthesis in Arabidopsis (Figure 8C).

## 4. Discussion

### 4.1. Melatonin and Photosynthesis

*SNAT1* appears to have originated from cyanobacteria according to the presence of a plant *SNAT1* ortholog in cyanobacteria [11]. Of note, *SNAT1* is present in the plastid genome of the red alga *Pyropia yezoensis*, whereas other *SNAT* genes from green algae and higher plants are present in their nuclear genome, suggesting possible endosymbiosis of cyanobacteria [12,50]. SNAT1 was first identified as an NSI with histone acetyltransferase activity [1]. Thereafter, it was found that SNAT1 displays both serotonin *N*-acetyltransferase activity in melatonin synthesis and protein lysine *N*-acetyltransferase activity [3,5]. SNAT1 plays a pivotal role in melatonin biosynthesis in various plant species. Suppression of *SNAT1* led to a decrease in melatonin, followed by enhanced pathogen susceptibility [6], salinity hypersensitivity [8], and susceptibility to high-light stress [7]. *SNAT1* OE increased melatonin levels, resulting in cadmium tolerance [51], delayed senescence [14], lateral root promotion [52], salt tolerance [53], and thermotolerance [16]. In addition to the genetic evidence, exogenous melatonin treatment conferred stress tolerance to a wide range of biotic and abiotic stressors, including viral attack [8,54,55]. In addition to being involved in defense responses, melatonin is also involved in plant growth in conjunction with photosynthesis when plants were challenged with abiotic stresses [20,56]. For example, melatonin treatment enhanced soybean growth by increasing PSI- and PSII-related genes and increased the rate of photosynthesis in Chinese hickory and grape seedlings upon stresses [57,58,59]. Melatonin treatment in tomato seedlings increased PSII activity and the photochemical quenching coefficient against salt stress [60]. Based on these findings, it is clear that melatonin is implicated in photosynthesis and growth, but its exact role in photosynthesis during normal growth and development conditions remains unknown thus far [61,62]. Based on our new data from the *snat1* mutant, we found for the first time that melatonin catalyzed by SNAT1 plays an important role in improving starch synthesis via the regulation of CPQC in plants.

### 4.2. Melatonin Signaling Pathway in CPQC

SNAT1 uses various substrates including histones, proteins, and serotonin. The *snat1* mutant grown under the light intensity of 50 μmol m^−2^ s^−1^ exhibited a severe defect in leaf size relative to that of 100 μmol m^−2^ s^−1^ light intensity (Figure 1 and Figure S1). Light intensity at 50 μmol m^−2^ s^−1^ did not induce low-light stress in Arabidopsis, whereas that below 30 μmol m^−2^ s^−1^ induced low-light stress [63]. The light-dependent dwarfism phenotype was also observed in transgenic *SNAT2*-knockout rice under low light (30 μmol m^−2^ s^−1^), whereas no such dwarfism was observed under high light (600 μmol m^−2^ s^−1^) [64]. The major cause of the *snat1* dwarfism phenotype in Arabidopsis was decreased starch accumulation, compared with the WT. This reduced starch accumulation in *snat1* was independent of the regulation of starch synthesis or degradation (Figure 2).

To achieve optimal photosynthesis or plastid biogenesis, chloroplasts require the CPQC system, consisting of CpHSPs and Clp, for correct folding of the many proteins imported from the cytoplasm [43,65,66,67]. Clp family members are ATP-dependent serine proteases that include many subunits, such as ClpR and ClpP. Among these Clp subunits, ClpR1 plays an important role in the accumulation of many chloroplast-localized proteins, including Lhcb2, RBCL, RBCS, Cpn60, and several PSI subunits [44]. Interestingly, *ClpR1* knockout Arabidopsis (*clpR1*) caused a marked decrease in the RBCL protein level but an increase in the *RBCL* mRNA level compared with the WT, suggestive of a pivotal role of ClpR1 for CPQC. Comparable with *clpR1*, *snat1* showed a significant decrease in the levels of ClpR1 protein and other chloroplast-localized proteins, including Lhcb1, Lhcb4, and RBCL. However, *snat1* showed a dramatic increase in the *RBCL* mRNA level, similar to that in *clpR1*, suggesting that SNAT1 is involved in CPQC.

Chloroplasts possess chaperone proteins, including various CpHSPs and Cnp60, which promote the correct folding and assembly of chloroplast-localized proteins. Among the chloroplast chaperones, two plastid HSP70 proteins, CpHSP70.1 and CpHSP70.2, have been well investigated in Arabidopsis [68]. Knockout of *CpHSP70.1* resulted in dwarfism, but knockout of *CpHSP70.2* resulted in a comparable phenotype with that of the WT. Mutants defective in both *CpHSP70.1* and *CpHSP70.2* are lethal, suggesting the crucial role of CpHSPs in CPQC [69]. In this study, the mRNA level of only two chaperones, *CpHSP70.1* and *CpHSP70.2*, were decreased in *snat1*. These results further indicate that SNAT1 is involved in the regulation of chloroplast HSP chaperones.

The correct balance between ROS production and scavenging in chloroplasts is essential for photosynthesis and plant growth; otherwise, oxidative damage can occur, leading to plant cell death [48]. Many antioxidants and antioxidant enzymes have roles in orchestrating ROS balance in chloroplasts. Of these, APX1 is a central player in chloroplast ROS regulation, as suggested by a defective chloroplast ROS-scavenging system, together with a late-flowering phenotype and stunted growth, in an *APX1*-knockout Arabidopsis mutant [48]. *GST* superfamily genes also participate in regulating ROS level balance and protecting plants from various oxidative stresses [70]. Suppression of the expression of *APX1* and *GST* genes was observed in *snat1*, suggesting the involvement of the ROS defense system of SNAT1.

Melatonin interacts directly with a variety of ROS, functions as a potent radical scavenger, and induces a series of antioxidant enzymes, including APX, superoxide dismutase, and catalase, in plants [17,71]. The radical scavenging and antioxidant enzyme induction functions of melatonin are believed to be mediated by a plant melatonin receptor (PMTR), but the integrity of a previously proposed PMTR is controversial [34,35]. In contrast to the PMTR, it is clear that melatonin-mediated defense responses against pathogen attack and ER stress require the MAPK cascade signaling, especially MPK3 and MPK6 [9,36].

## 5. Conclusions

In this study, we showed that suppression of starch accumulation in *snat1* was mediated by SNAT1-catalyzed melatonin decrease, which triggers the decreased accumulation of Lhcb1, Lhcb4, and RBCL proteins. The suppression of these photosynthesis-related proteins was ascribed to the combined effects of both CPQC (*CpHSP70s* and ClpR1) and ROS defense system (*APX1* and *GSTF6*) (Figure 8C). Of note, the expression of *ClpR1*, *APX1*, and *GSTF6* was partly dependent on the MAPK pathway.

Based on our data, we can conclude that defective *SNAT1* expression gives rise to dwarfism and a delayed flowering phenotype, and the causative factor of these defects is melatonin, which is catalyzed by SNAT1.

## Data Availability

The data that support the finding of this study are available from the corresponding author upon reasonable request.

## Supplementary Materials

The following are available online at https://www.mdpi.com/2076-3921/10/4/511/s1, Figure S1: Phenotypic difference depending on light intensity in Arabidopsis wild type, *snat1*, and OE at four weeks after planting, Table S1: Sequences of primers in quantitative real-time RT-PCR.
