# Supplementary material for: Melatonin Regulates Chloroplast Protein Quality Control via a Mitogen-Activated Protein Kinase Signaling Pathway
Source: Antioxidants (Basel). 2021 Mar 25;10(4):511. doi: 10.3390/antiox10040511 (PMC8064490; doi:10.3390/antiox10040511)
Supplement: Supplementary file 1 [file antioxidants-10-00511-s001.pdf]

Fig. S1 Phenotypic difference depending on light intensity in Arabidopsis wild type, *snat1*, and OE at 4 weeks after planting.

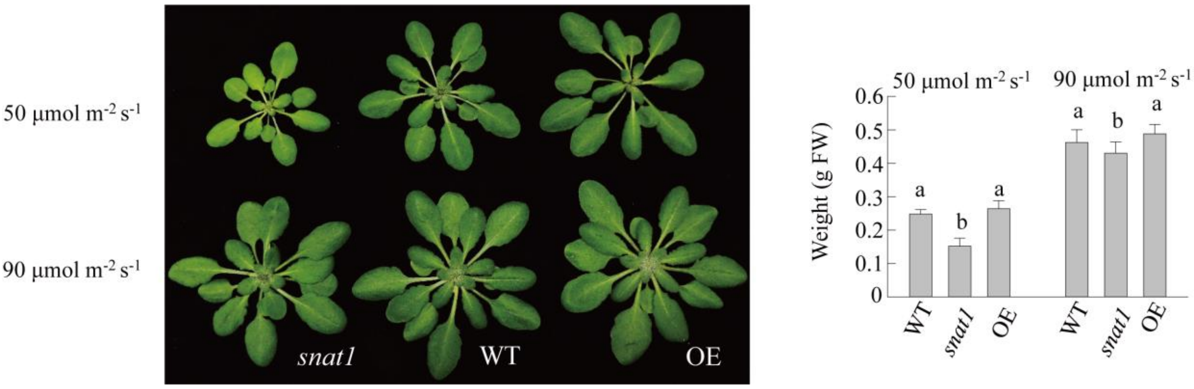

Supplemental Table 1. Sequences of primers in quantitative real-time RT-PCR

| Gene | Forward (5´-3´) | Reverse (5´-3´) |
|---|---|---|
| GST1 | GGACTCACCAAGCCTGTGTT | TGAATCGCATGAGTTTGACC |
| GST6 | CCCAGCCATCCATTACTTGT | TCGTTTATTTGGGCAAAAGG |
| GSTF9 | CTCACCTCCCGTTCACTGAT | CACGAGGGTGGCAACATTACA |
| APX1 | GTGTGTGTCTCCCCGAGAGT | CGAAGCAGCAAAAGCGCAAC |
| CpHSP70.1 | GGAAGTGGATCAACCCAAGA | GCATCGATCACATCATCACC |
| CpHSP70.2 | TTCGTCTTCTTCCTCCTCCA | GTATCGGGAAGCGTTGTTGT |
| ClpR1 | CAATCCCTAATTTCGCCTCA | TGGAACAGACAGCTTCATGG |
| ClpR4 | TCAGCGATTATCCCGTCTTC | CGTTTAGGCTTGGAACTGGA |
| ClpP1 | TTCGAAGTCCTGGAGAAGGA | TTACCCATCCACCAGGAGAG |
| Lhcb1.1 | AGCCATCGTCACTGGTAAGG | CCATCCACCACAAACACAAA |
| Lhcb1.3 | ACTTTGTTCCCGGAAAGTGA | TCTCCGAAGGACATAAAACGA |
| Lhcb1.4 | GGCATTGACGATATCACGAG | GTGAGAGTGTGGCGCAAGTA |
| Lhcb4.1 | CACTCCACACCACCATCATC | TCATGGGCCAAACATGTAAA |
| RBCL | GTGTTGGGTTCAAAGCTGGT | CATCGGTCCACACAGTTGTC |
| RBCS1A | CACAAAGAGTAAAGAAGAACA ATGGC | AGATGGGGGATAAAGTTTTGA GG |
| RBCS3B | CACCAGTAGGAAAACAAGTCA GTAAG | CAGACACAGACAATAGGAAAT GAAATGAGCAGAG |
| sAPX | CGTTATCTAATGGGGGCACA | GAAAGCCCAAAACCATGAAA |
| tAPX | GGAAGCCCAGATAAGCCATT | TGATGTTTTTGTTTGCAAGG |
| Hsp90c | CAAAAGCGGAGAAACAGAGG | TCATTAGGCCCATGATACTGC |
| ARC2 (cpn60) | CACTCAGGCCATTGTTGTTG | TTGCAGATAAATGGGGATGA |
| DWF4 | CAAAGCCATTAGAAAGAGAAA GTGA | CAAATTTTTATATATCATTGG GCAAA |
| BZR1 | GAAGTTTCGAGGGGTTGGTT | CGCAGCTACAGCTCTTCTCC |
| CDC2b | CCCATATTTTGACAGCCTTGA | CATCTCATTGCTACCACACCA |
| KS | CCAAGTTGATCTGGCAGGTA | TTGTCTCCTAAAATCAATTTT CCTC |
| IAA1 | TGGACGGAGCTCCATATCTC | ATCACCGACCAACATCCAAT |
| EXP1 | GAGTGCTTGATTTTGCACGA | CGCTTCGAGAAGGGATACAA |
| SEX1 | TGTACGTTACGTTTTCTGTCCA | CATATCGCTCGACCAGTGAA |
| PGM1 | AAACCGAGGAGGAGGAAAAA | CGTGTAGGTCGACGTCATTG |
| EF-1a | TGGTGACGCTGGTATGGTTA | CATCATTTGGCACCCTTCTT |
